# Extraordinary Development of the Mammary Glands

**Published:** 1858-12

**Authors:** 


**EXTRAORDINARY DEVELOPMENT OF THE MAMMARY GLANDS.**

We learn from the *Gazette Hebdomidaire* (Oct.) that there is in the wards of M. Manec LaCharite Hospital, Paris, a case of extraordinary development of the Mammary Glands.

The subject is a delicate female only seventeen years old. Each Gland weighs (as near as can be estimated) from sixteen to eighteen pounds, and when the woman is in the erect position, reaches the pubis.

The circumference of the Glands at their base, measures twenty-four inches, and at the largest point, one is thirty-three and the other thirty-six inches.

This rare development, which is regarded by M. Manec and others as a hypertrophy of the entire organ, gland, cellular tissue and skin, made its appearance about two years ago without any apparent cause.

She menstruated for the first time a year after the commencement of the enlargement, but has never been regular. For the past eight months, this function has entirely ceased.

Complete ablation of the entire mass is the treatment suggested.

